# Effect Assessment of Aurantio-Obtusin on Novel Human Renal Glomerular Endothelial Cells Model Using a Microfluidic Chip

**DOI:** 10.3390/nu14214615

**Published:** 2022-11-02

**Authors:** Wen Qin, Zhuo Yang, Jiyong Yin, Di Chen, Junsheng Huo, Jingbo Wang, Liyuan Wang, Qin Zhuo

**Affiliations:** 1 Department of Central Laboratory, National Institute for Nutrition and Health, Chinese Center for Disease Control and Prevention, Beijing 100050, China; 2 Department of Food Science and Technology, National Institute for Nutrition and Health, Chinese Center for Disease Control and Prevention, Beijing 100050, China

**Keywords:** microfluidic chip, aurantio-obtusin, human renal glomerular endothelial cell, nephrotoxicity, visualization

## Abstract

Cassiae semen is widely used as a raw material of health food. Anthraquinone compounds, the main components in cassiae semen, have been reported to show nephrotoxicity. Aurantio-obtusin (AO) is a major anthraquinone compound extracted from cassiae semen. This study investigates the effects of AO on the morphology and physiological function of human renal glomerular endothelial cells (HRGECs) on a microfluidic chip device for the first time. HRGECs were cultured on a microfluidic plate and exposed to a series of AO concentrations. Compared with traditional 96-well culture, HRGECs cultured on the microfluidic chip appeared to better mimic the glomerular microenvironment in vivo. AO induced different degrees of damage to cellular morphology and physiological function. The leakage of lactate dehydrogenase (LDH), as well as the secretion of interleukin-6 (IL-6), tumor necrosis factor-α (TNF-α), transforming growth factor-β1 (TGF-β_1_), and monocyte chemoattractant protein 1 (MCP-1), increased in the AO treated groups. At the same time, cell viability and expression of ZO-1 in the AO treated groups decreased in a dose-dependent manner. The innovative device enables direct visualization and quantification to evaluate the cytotoxic effects of AO on HRGECs, and provides a useful visual in vitro model for studying health effect of health food.

## 1. Introduction

The kidney is composed of more than 20 cell types with different ultrastructures, metabolic capacity, and transport function. When chemicals are excreted through the kidney, they are selectively enriched in renal cells, potentially causing damage to the cell membrane, mitochondria, endoplasmic reticulum, and lysosomes, disrupting cell integrity and causing apoptosis or necrosis of renal cells [1,2]. The necrotic cells enter the lumen of the renal tubules, leading to blockage and obstructing the flow of urine, thereby reducing the filtration and reabsorption rate of the glomeruli, thus resulting in an imbalance of water and electrolytes in the body. In severe cases, this can cause acute renal failure. The ability of the kidney to filter, secrete, and reabsorb is extremely important for maintaining homeostasis and it is similarly vulnerable to drug toxicity [3].

Accordingly, studies on drug nephrotoxicity are an essential part of drug safety evaluation. At present, the main methods to evaluate the nephrotoxicity of drugs are mostly based on in vitro cytotoxicity assays or in vivo evaluation in experimental animals. Conventional in vivo animal tests are time-consuming, costly, and cannot fully mimic the human body. Accordingly, in vitro models of renal cells are widely used in drug nephrotoxicity studies due to their long survival time in vitro and ease of subculture, as well as their rich metabolic transformation and biotransformation functions [4,5].

However, these in vitro cell-based models cannot recapitulate the biological responses of humans, since the cells grown in vitro lost the neurohumoral regulation and interaction between cells. Growing in a relatively static environment, the cells do not function as they do in vivo except for proliferation, nor can they truly reflect the survival situation in vivo [6].

In recent years, the advances of microfluidic chip technology provide an excellent solution to simulate the in vivo physical environment of human organs. Specifically, microfluidic organ chips can immediately adjust the microenvironment associated with cell growth and function in accordance with cell type, while the flowing medium can supply adequate nutrients and exclude harmful metabolic wastes from the periphery of the cells [7]. In addition, the chip regulates the oxygenation levels, provides shear forces, and maintains the barrier function of the cell layer [8]. Using an optimized microfluidic device, we could clarify and understand the unfavorable toxic effects and prevent them from occurring in human clinical trials. Recently, researchers tried to study the renal tubule system using a microfluidic device to simulate the toxicity of various compounds. For example, Jang et al. described a microfluidic device that exposes human renal epithelial cells to the flowing fluid in a certain arrangement to mimic the original functions of the human renal proximal tubule. Compared with traditional cell models, cisplatin toxicity and P-glycoprotein (Pgp) efflux transporter activity measured in the chip-based model were closer to the results of in-vivo experiments [9]. Additionally, Sakolish et al. demonstrated a reusable microfluidic model of the human proximal tubule, in which the renal epithelial cells grew in various states of four representative renal disease. Compared with traditional static cultures, the growth of cells in the microfluidic device potentially enabled more realistic and accurate predictions of organismal responses [10]. However, these cell-based models mainly focused on the renal tubule system, and there are few studies on in vitro glomerular models on microfluidic chips for evaluating drug nephrotoxicity. Glomerular filtration is the first step in the formation of urine, and it constitutes the basic physiological function of the kidney. When the glomerular filtration barrier is damaged, many chronic kidney diseases occur. Glomerular endothelial cells are the main cells that constitute the glomerular filtration barrier, and their condition greatly affects the normal function of the barrier. Therefore, it is worthwhile to construct an in vitro glomerular cell model to evaluate drug nephrotoxicity.

Anthraquinone compounds are the effective substance of many traditional Chinese medicinal herbs, such as rhubarb, cassiae semen, *Polygonum multiflorum*, and senna leaf [11]. These compounds have many physiological effects, such as purgative, liver clearing, lipid lowering, bacteriostatic activities. However, the side effects of anthraquinone-containing traditional Chinese health products represented by *Polygonum multiflorum* have been reported frequently in recent years, and their potential safety problems have attracted increasing attention [12,13,14].

Cassiae semen, mature seeds of the leguminous plant *Cassia obtusifolia* L. or *C. tora* L., are one of 110 traditional Chinese medicinal herbs with homology of medicine and food registered by the National Health Commission of China. Anthraquinones are not only the pharmacodynamic basis of cassiae semen, but also the main factors that may cause safety risks. At present, there are few reports on the safety of cassiae semen for consumption in China and abroad. Previous animal experiments showed that anthraquinones in cassiae semen have strong negative effects on the kidney, liver, digestive and reproductive systems [15,16]. AO is a unique anthraquinone compound extracted from cassiae semen with antihyperlipidemic and anti-inflammatory effects [17,18]. It is used as a quality control standard and a detection index for the identification of cassiae semen, as stipulated by the Chinese Pharmacopoeia [19]. In this study, we chose the specific anthraquinone of cassiae semen to study the nephrotoxicity of AO in a microfluidic device lined with human renal glomerular endothelial cells (HRGECs). Cell viability, LDH leakage, IL-6, TNF-α, TGF-β_1_, and MCP-1 secretion, as well as the expression of the tight junction protein ZO-1 in HRGECs were examined under AO exposure. The permeability of the HRGEC layer in a transwell chamber was also assessed. To our best knowledge, this is the first attempt to use HRGECs in a microfluidic device for a study of AO-induced nephrotoxicity. This model has the potential to become an efficient visual platform for evaluation of raw materials of health food as a complement to animal models.

## 2. Materials and Methods

### 2.1. Cells and Culture Media

**Figure 1:**
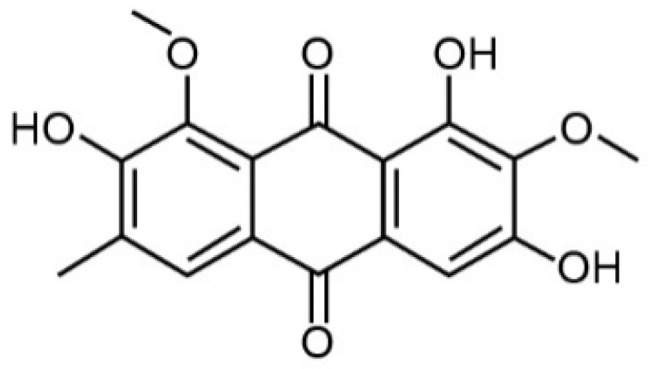
Structural formula of Aurantio-obtusin.

Human renal glomerular endothelial cells (HRGECs) were purchased from BNCC (Beijing, China), obtained from ScienCell (catalog no. 4000, San Diego, CA, USA), and AO was purchased from NIFDC (Beijing, China), with a purity of 99% (structural formula in Figure 1). Endothelial cell medium (ECM), 1% penicillin–streptomycin, and 5% Endothelial Cell Growth Supplement were purchased from ScienCell (San Diego, CA, USA). Fetal bovine serum (FBS) and 0.25% Trypsin-EDTA were purchased from Gibco (New York, NY, USA).

### 2.2. Cell Culture

HRGECs were cultured in ECM containing 5% FBS, 1% penicillin–streptomycin solution and 5% Endothelial Cell Growth Supplement in the cell incubator with 5% CO_2_ at 37 °C.

### 2.3. Culturing HRGECs on a Microfluidic Chip

**Figure 2:**
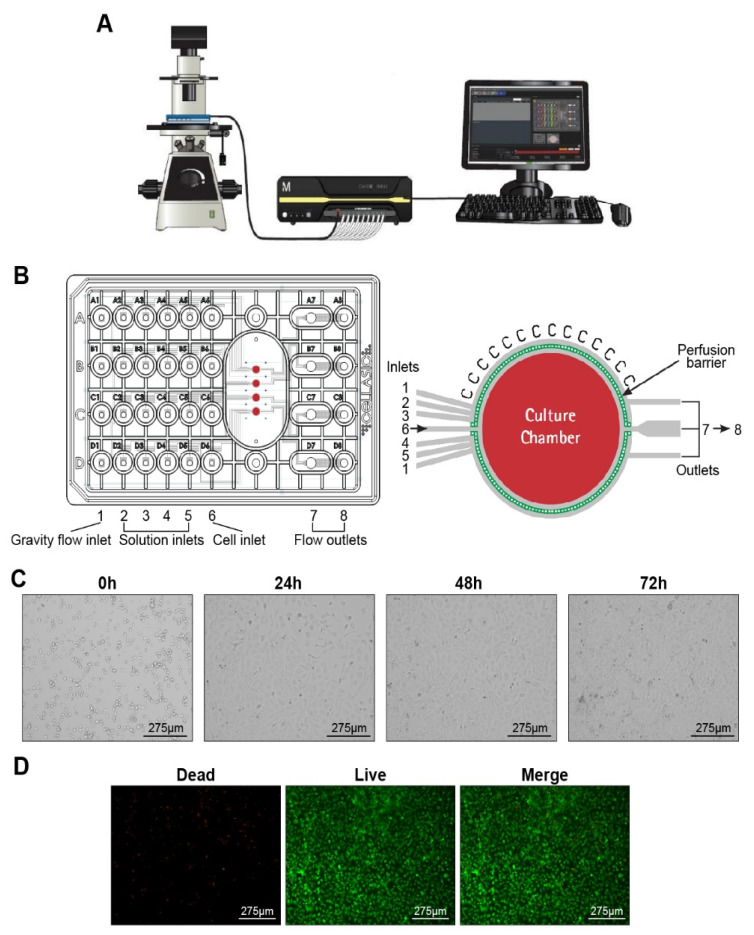
Design of a CellASIC-based microfluidic platform to culture glomerular endothelial cells. (**A**) CellASIC ONIX2 Microfluidic Platform. The Platform consists of a controller system including multiple control panels, a computer software interface and a fluorescent inverted microscope. (**B**) Configuration of the microfluidic chip device. The microfluidic plate consists of four independent culture units, each with a gravity flow inlet (1), four solution inlets (2–5), and a cell inlet (6). Each row of wells addresses the corresponding culture chamber. (**C**) Bright-field images showing that HRGECs grew on the microfluidic chip at different culture times (0, 1, 2, 3 d). (**D**) Fluorescence images of live/dead staining of HRGECs after 3 days of culture on the chip.

The CellASIC^®^ONIX2 Microfluidic Platform and the M04S microfluidic plate (Merck Millipore, Hayward, CA, USA) were used for cell culture. The Microfluidic Platform consisted of a controller system including multiple control panels, a computer software interface and a fluorescent inverted microscope (Figure 2A). The M04S microfluidic plate has four independent culture units, each with a gravity flow inlet (1), four solution inlets (2–5), a cell inlet (6), and two outlets (7 and 8) (Figure 2B).

HRGECs cultured for 3–5 days were digested with 0.12% trypsin for 2–3 min, centrifuged and resuspended with culture medium to a cell concentration of 2 × 10^6^ cells/mL. Then, 10 µL of the cell suspension was added to well 6 of the M04S microfluidic plate, and 350 µL of prepared ECM medium was pipetted into well 1. The microfluidic plate was fixed to the ONIX2 manifold, and evacuated to create a hermetic environment. The pressure and flow time for well group 6 were 0.25 psi and 8 s when loading cells. After loading, the cells were cultured at 37 °C and 5% CO_2_, and the culture medium in well 1 continued to flow over the cells by gravity at a flow rate of about 5 µL/h, enabling the cells to grow adherently in the culture chamber.

### 2.4. Evaluation of AO-Induced Cytotoxicity in HRGECs

In order to assess the nephrotoxicity of AO, HRGECs were exposed to a series of AO concentrations (0, 50, 100, and 200 µM) for 48 h. The AO stock solution was prepared by dilution with dimethyl sulfoxide (DMSO) to 30 mM, and then diluted in culture medium to the indicated final concentrations. The cell culture medium containing AO (0, 50, 100, and 200 µM) was pumped from well 2 into the culture chamber, whereby the pump 2 pressure was set at 0.8 psi and flow rate was approximately 6 µL/h. After two days of drug exposure, the effluent was collected from well 7 and 8 for LDH, IL-6, TNF-α, TGF-β_1_ and MCP-1 detection. The assays were performed as described in the instructions of the LDH Assay Kit (Jiancheng, Nanjing, China), Human IL-6 ELISA Kit, Human TNF-α ELISA Kit, Human TGF-β_1_ ELISA Kit and Human MCP-1 ELISA Kit (Beyotime, Shanghai, China).

After 2 days of drug treatment, apoptosis was detected using the Live and Dead Viability/Cytotoxicity Assay Kit (KeyGEN BioTECH, Nanjing, China). The adherent cells in the culture chamber were gently washed with PBS, after which the prepared fluorescent dye continued to flow through the culture chamber at a flow rate of about 35 µL/h, and allowed to stain the cells at room temperature for 30 min. Living cells exhibited green and dead cells red fluorescence. Intracellular fluorescence was quantified via fluorescence microscopy.

Cell viability was assessed using the Cell Counting Kit-8 (Dojindo, Japan). An aliquot comprising 10 µL of CCK-8 reagent in 100 µL medium was added to the solution inlets, and allowed to flow continuously through the culture chamber at a flow rate of 35 μL/h under 3 psi pressure. The effluent from wells 7 and 8 was collected after 3 h, and the absorption at 450 nm was measured.

### 2.5. Real-Time Quantitative PCR

The cells in the culture chamber were digested with trypsin, centrifuged to collect the cell precipitate, and the cells were lysed by adding lysis solution. Total RNA was extracted by using RNAprep pure Micro Kit (TIANGEN, Beijing, China), and then reverse-transcription and Real-time fluorescence quantitative PCR was conducted through the LightCycler 480 system (Roche Diagnostics, Rotkreuz, Switzerland) according to the instructions of FastKing One Step RT-qPCR Kit (TIANGEN, Beijing, China). Reverse-transcription was performed at 50 °C for 30 min, then it was denatured at 95 °C for 3 min, and the following cycling conditions were used: 95 °C, 15 s and 60 °C, 30 s for 40 cycles. The primers for amplification of ZO-1 and GAPDH were in Table 1. The relative expression of mRNA was calculated using 2^−ΔΔCt^ method and normalized to the expression of GAPDH.

### 2.6. Immunofluorescence Assay

The effect of different concentrations of AO on the expression of tight junction protein ZO-1 in HRGECs was assessed by immunofluorescence staining. After 2 days of drug treatment, immunofluorescence staining for ZO-1 was performed. The cell samples were gently washed with PBS for 15 min, and then fixed in 4% paraformaldehyde for 30 min at room temperature. The fixed cells were washed for 15 min with PBS and blocked with 5% goat serum (Solarbio, Beijing, China) for 25 min at 37 °C, incubated at 37 °C for 2 h with the ZO-1 primary antibody (diluted at 1:50, Abcam, MA, USA, washed with PBS for 15 min, incubated with the secondary antibody (diluted at 1:400, Beyotime, Shanghai, China) for 1 h at room temperature, washed with PBS for 15 min, and counter-stained with DAPI (Sigma Aldrich, St. Loius, MO, USA) for 10 min in the dark. The pressure was set at 2 psi. All of the images were recorded under a fluorescence microscope.

### 2.7. Permeability Testing of the HRGEC Layer

The glomerular endothelial cells were seeded into a transwell chamber (Corning, New York, NY, USA) to study the barrier permeability of the HRGEC layer. The cells were cultured in prepared ECM medium and seeded into the culture plate inserts at a density of 1 × 10^5^ cells/mL. The cells grew on 0.4-µm polycarbonate membranes in the inner chamber, while 0.5 and 1.5 mL of prepared ECM medium was added to the inner and outer chamber, respectively. The cells were cultured at 37 °C with 5% CO_2_ for 24 h, after which the supernatant was discarded and 0.5 mL cell culture medium containing AO (0, 25, 50, 100, and 200 µM) was added to the inner chamber. After treatment with AO for 48 h, the supernatant in the inner and outer chambers was aspirated and the cells were gently washed with PBS, followed by the addition of 0.5 mg/mL FITC-IgG (Solarbio, Beijing, China) in 0.5 mL PBS to the inner chamber and 1.5 mL culture medium to the outer chamber. The filtrates in the outer chamber were collected and replaced with 1.5 mL culture medium at 15, 30, 45, and 60 min. The blank control with no cells underwent the same procedure as above. The fluorescence values of the collected filtrates were recorded using a fluorometer (Molecular Devices) at an excitation wavelength of 485 nm and emission wavelength of 510 nm.

### 2.8. Statistical Analysis

All experiments were performed in triplicate, and data were presented as means ± standard deviations. One-way analysis of variance (ANOVA) with a Tukey–Kramer multiple comparisons test was performed using GraphPad Prism 8.0 (GraphPad Software Inc., San Diego, CA, USA). Differences between groups were considered statistically significant at *p* < 0.05.

## 3. Results

### 3.1. Establishment of a Microfluidic Glomerular Model Using HRGECs

The CellASIC ONIX2 Microfluidic Platform and M04S Plates were utilized to culture glomerular endothelial cells. The cell suspension comprising 2 × 10^6^ cells/mL was perfused into the cell chamber pre-coated with the extracellular matrix containing collagen type I. The culture medium continued to flow over the cells at a flow rate of about 5 µL/h, and provided sufficient nutrition and minimum pressure close to microenvironment of cells in vivo. The growth state of the cells in the culture chamber was observed at different times, as shown in Figure 2C. The cells began to grow adherently at approximately day 1, and their morphology varied between round, triangular, fusiform, and irregular. At day 2, the confluence ratio of the cells was greater than 80%. As the incubation time increased, the cells were able to proliferate normally to form a dense cell layer at day 3. The results of live/dead cell staining showed that a well-defined confluent cell monolayer was formed, HRGECs proliferated well in cell chamber (Figure 2D).

### 3.2. Assessment of AO-Induced Cytotoxicity in Glomerular Endothelial Cells

**Figure 3:**
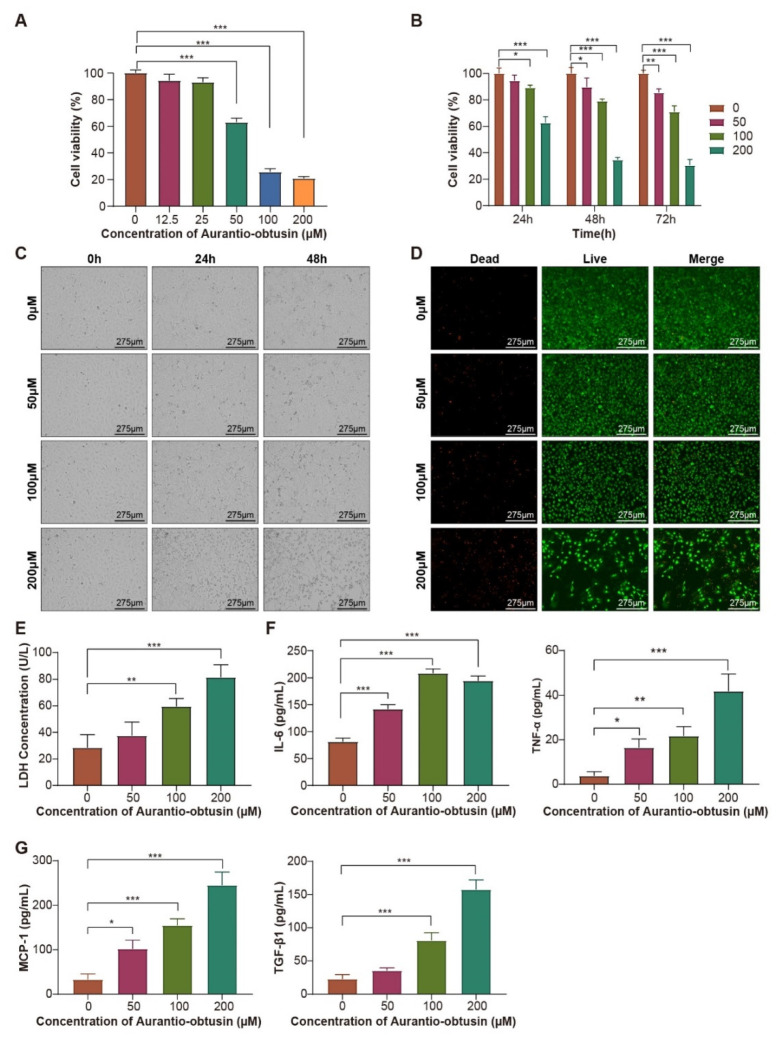
Cytotoxic effects of AO on glomerular endothelial cells. (**A**) CCK-8 assay of HRGEC viability in 96-well plates. (**B**) CCK-8 assay of HRGECs viability on the microfluidic chip. (**C**) Morphological changes in HRGECs after exposure to AO on the microfluidic chip were observed using a bright-field microscope. (**D**) Fluorescence images of live/dead staining of HRGECs on the chip after exposure to AO. The live cells were stained green and the dead cells were stained red. (**E**) LDH leakage assay in HRGECs. (**F**) AO treatment increased the expression of pro-inflammatory cytokines IL-6 and TNF-α. (**G**) AO treatment increased the expression of cytokines TGF-β_1_ and MCP-1. The data are presented as means ± standard deviation. *n* = 3, * *p* < 0.05, ** *p* < 0.01, *** *p* < 0.001.

We performed a pre-experiment to assess the toxic effects of AO on HRGECs in 96-well plates. HRGECs were treated with different concentrations of AO (0, 12.5, 25, 50, 100, and 200 µM), and the cell viability was detected using the CCK-8 assay 48 h later. The cell viability showed a gradual decrease with the increase in AO concentration, and when the AO concentration reached 50 µM, the cell viability was significantly lower than that of the control group (*p* < 0.001, Figure 3A). Therefore, the AO concentration range of 50, 100, and 200 µM was selected for further experiments. At the same time, we detected the cytotoxicity of different concentrations of DMSO (0.17%, 0.33%, and 0.67%, corresponding to the concentration of DMSO in 50, 100, and 200μM AO solutions). The results showed that there was no significant difference in the survival rate of HRGECs treated with different concentrations of DMSO solution compared with blank control group. Thus, DMSO at the concentrations used had no effect on cell viability.

The main objective of constructing a human glomerular endothelial cell model on a microfluidic platform is to effectively predict human nephrotoxicity. We assessed cell viability when HRGECs were exposed to AO at different points in time. Cell viability decreased in a dose-dependent manner, as shown in Figure 3B, and similar trends were observed when the AO action times were extended from 48 to 72 h. Therefore, the AO action time was selected as 48 h for further experiments.

To assess the effects of AO on HRGECs, we also assessed LDH leakage, as well as IL-6, TNF-α, TGF-β_1_, and MCP-1 expression after exposure to AO at different concentrations (0, 50, 100, 200 µM) for 48 h on the microfluidic plate. In this model, it was possible to use a fluorescence microscope to observe changes in cell state in real time. When the AO concentration reached 100 μM, the morphology of cells began to change in a time-dependent manner, and some cells began to shrink (Bright-field, Figure 3C). Most of the cells appeared shrunken and fragmented following exposure to 200 μM AO for a prolonged time.

The proportion of dead cells increased gradually as the AO concentration increased (Figure 3D). LDH leakage, a classic marker of endothelial cell injury was also assessed. LDH leakage in the effluent significantly increased with increasing AO concentration (*p* < 0.01, Figure 3E), indicating significant biochemical changes in HRGECs after exposure to AO.

The secretion levels of IL-6 were significantly higher in AO-treated glomerular endothelial cells than in untreated controls (*p* < 0.05, Figure 3F), while high doses of AO had a strong cytotoxic effect, a decrease in IL-6 content was observed. The secretion levels of TNF-α, TGF-β_1_, and MCP-1 increased with increasing AO concentration (Figure 3F,G). The results above indicated that AO exposure induced an inflammatory response in the treated HRGECs.

### 3.3. Expression of the Tight Junction Protein ZO-1

**Figure 4:**
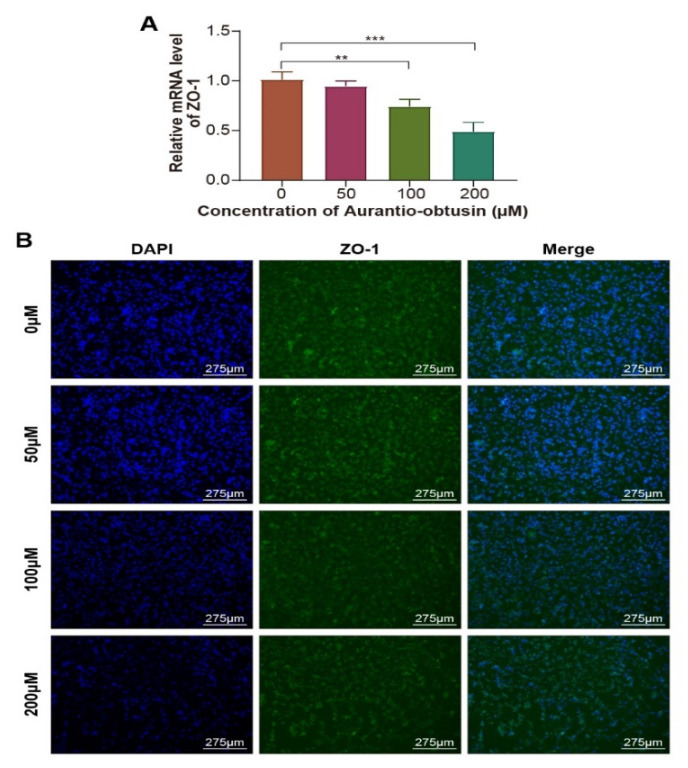
Expression of ZO-1 in HRGECs after exposure to AO. The cells were treated with AO (50 to 200 µM). (**A**) Real-time quantitative PCR analysis. (**B**) Immunofluorescence staining analysis. ZO-1 (green), DAPI (blue). The data are presented as means ± standard deviation. *n* = 3, ** *p* < 0.01, *** *p* < 0.001.

The tight junction protein ZO-1 expressed by glomerular endothelial cells plays an important role in the maintenance of glomerular filtration barrier function. We examined the effect of AO on the expression of ZO-1 in endothelial cells using a real-time quantitative PCR, as shown in Figure 4A, as AO concentration increased, the transcription level of ZO-1 gene decreased. Moreover, immunofluorescence assay was used to measure ZO-1 expression in HRGECs in response to AO. As shown in Figure 4B, AO exposure reduced the expression of ZO-1 in HRGECs compared with the control group. These indicated that the junction between cells became loose as the AO concentration increased, confirming that AO may disrupt barrier integrity by reducing the expression of ZO-1.

### 3.4. Effect of AO on Glomerular Endothelial Barrier Permeability

**Figure 5:**
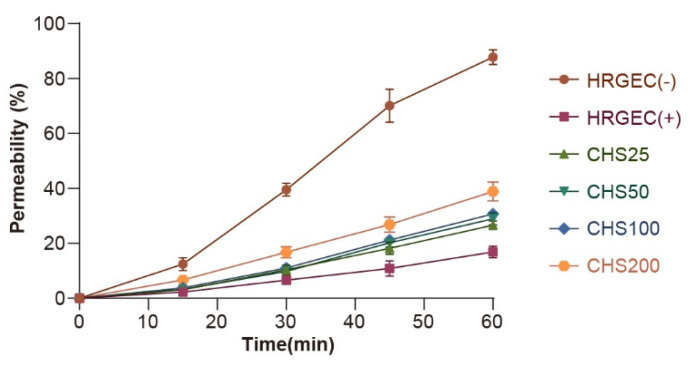
Effect of AO on the permeability of the glomerular endothelial barrier in a transwell chamber. The cells were treated with AO at different concentrations (25 µM to 200 µM) for 48 h, and the permeability of the glomerular endothelial barrier to FITC-IgG was measured within 60 min.

As a key feature of renal function, glomerular filtration is a very important observation index in the study of nephrotoxicity. In order to evaluate the effects of drug exposure on the permeability of the glomerular endothelial cell barrier, we studied the effects of AO on the permeability of cell monolayers for large molecules using a transwell chamber. The results showed that the permeability of the barrier to FITC-IgG increased after exposure to AO at different concentrations (25, 50, 100, and 200 µM) (Figure 5). This indicates that AO exposure caused different degrees of damage to the integrity of the cell monolayers.

## 4. Discussion

Drug nephrotoxicity affects the intracellular and extracellular environment, causing changes in cell proliferation, metabolism, physiological functions, and other aspects, which often lead to changes in cell morphology and eventually cell death [20]. The most direct and reliable method to detect cellular damage is by observing changes in cell morphology and structure using optical microscopy. The structural changes in cells caused by drug toxicity mainly include nuclear pyknosis, nuclear fragmentation, cell swelling, apoptosis, or necrosis. In this study, HRGECs were cultured in a microfluidic platform, which allowed real-time morphological observations under an inverted fluorescence microscope. These observations revealed cell contraction and apoptosis as the AO concentration increased. Compared with cells in the untreated control group, cells treated with 200 µM AO exhibited significant differences. The results of live/dead staining also showed that the number of dead cells increased greatly when the AO concentration reached 100 µM or above. Based on the results of cell morphology observation and CCK8 cell viability test, we have preliminarily confirmed that AO at concentrations less than 50 µM are relatively safe for HRGECs.

Cell migration, differentiation, proliferation, and apoptosis are largely regulated by their microenvironment, which is highly complex and dynamic. Microfluidic chips can accurately regulate the microenvironment of cells in time and space through precise microfluidic control technology. Applying stimulation with concentration gradients of different biochemical factor and stress to cells is helpful to identify the regulatory factors affecting cell biological behavior, so as to obtain specific cell responses and more reliable results [21]. In this study, we used 96-well plates and microfluidic chips to assess the cytotoxicity of AO. When the 96-well plate was used for static culture of cells, the cell viability decreased to 25.8% when the AO concentration reached 100 µM, while in the microfluidic chip, the cell viability was 79.0% when the AO concentration reached 100 µM. It can, therefore, be seen that the cell viability under the microfluidic dynamic culture conditions was significantly higher than under the static culture condition. This may be due to the static microenvironment, where nutrient depletion and accumulation of metabolic wastes around cells inhibits cell growth. By contrast, microfluidic dynamic culture provides a constant supply of nutrients and oxygen, while also reducing the accumulation of metabolic wastes. In addition, fluid motion can reproduce various types of mechanical signals caused by physiological flow (e.g., blood and interstitial flow) and tissue deformation (e.g., respiration, peristalsis, and heartbeat), including fluid shear stress, tension, etc., while the low flow rate of 5 µL/h can reduce the cell damage caused by fluid shear stress [22,23]. Compared with traditional static culture, HRGECs cultured on the microfluidic chip appear to better mimic the complex pathophysiology associated with drug toxicity in the human kidney.

Lactate dehydrogenase (LDH) leakage is an indicator of cell membrane damage [24], and various studies have confirmed that drug-induced renal injury causes an increase in the release of this enzyme [3,20,25]. Our results showed that LDH leakage increased in a dose-dependent manner after treatment with 50 to 200 µM AO, indicating that the cell membrane integrity was disrupted following exposure to AO.

The induction of IL-6 has been observed during the development of acute kidney injury (AKI) in humans and experimental animals alike [26,27], and IL-6 is produced in different amounts by endothelial cells in response to proinflammatory signals such as TNF-α [28]. TGF-β_1_ and MCP-1 were also induced when glomerular injury and inflammation occurred [29,30]. In vitro and in vivo experimental studies [31,32] have found that the inflammatory cytokine TNF-α can induce the production of reactive oxygen species (ROS), and further promote oxidative stress and inflammation in the process of renal disease. ROS can activate the production of transcription factors as NF-κB and AP-1, and induce the production of TGF-β_1_ and MCP-1. In addition, MCP-1 can promote glomerular fibrosis through TGF-β_1_ and NF-κB pathways. These cytokines interact and restrict each other through autocrine and paracrine pathways, forming a complex cytokine network in the process of kidney injury and disease, and eventually leading to renal function injury. Therefore, we selected the above cytokines to evaluate the toxic effect of AO on HRGECs. In this study, we found intermediate concentrations of AO induced the production of the inflammatory cytokine IL-6 in HRGECs, but the IL-6 content decreased when the AO concentration increased to 200 µM. This may be due to the prolonged exposure to high concentrations of AO that accentuated its toxic effects on HRGECs, increasing apoptosis and thereby blocking the inflammatory response. By contrast, AO also induced the secretion of TNF-α, TGF-β_1_, and MCP-1 at high concentrations. The trends of secretion were different from IL-6, which may be due to the different regulatory networks controlling the cytokines. Further studies are needed to explain it well.

ZO-1 is a tight junction protein that plays an important role in regulating cellular material transport, maintaining cell permeability, promoting cell proliferation, and differentiation, as well as regulating and maintaining the epithelial barrier. In STZ-induced diabetic nephropathy rat models, the expression of ZO-1 was found to be closely related to the incidence of proteinuria [33]. Li et al. studied the effects of Cd^2+^ on glomerular endothelial cells on microfluidic chips, and found that Cd^2+^ exposure clearly reduced the expression of ZO-1 in glomerular endothelial cells compared with the control group [25]. In this study, AO reduced the expression of ZO-1 in HRGECs, which indicated that AO may disrupt barrier integrity by reducing the expression of tight junction proteins. The results of permeability testing of the HRGEC layer showed that the permeation rate of fluorescence-labeled IgG across cell layer gradually increased with the increase in AO concentration, which may be due to the shrinkage of HRGECs after the decrease in tight junction protein expression, leading to the increased permeation of large molecules through the glomerular endothelial layer.

## 5. Conclusions

In the study, we examined the nephrotoxicity of AO using a model based on HRGECs cultured on a microfluidic chip. The results showed that AO caused various degrees of damage to HRGECs, as indicated by changes in physiological function and cellular morphology. Our HRGEC-based microfluidic model facilitates the evaluation of AO-induced nephrotoxicity in a physiologically relevant microenvironment, and offers a potential alternative to traditional cell culture methods for safety evaluation of health food raw materials.

## Figures and Tables

**Table 1:** Primers used for RT-qPCR analysis.

Gene	Forward	Reverse
GAPDH	5′-ACAGTCAGCCGCATCTTCTT-3′	5′-GTTAAAAGCAGCCCTGGTGA-3′
ZO-1	5′-GCGGTCAGAGCCTTCTGATC-3′	5′-CATGCTTTACAGGAGTTGAGACAG-3′

## Data Availability

The data supporting reported results can be obtained from the corresponding author.
